# The Impact of Time to Initiate Therapeutic Hypothermia on Short-Term Neurological Outcomes in Neonates with Hypoxic–Ischemic Encephalopathy

**DOI:** 10.3390/children11060686

**Published:** 2024-06-04

**Authors:** Till Dresbach, Viktoria Rigoni, Anne Groteklaes, Thomas Hoehn, Anja Stein, Ursula Felderhoff-Mueser, Andreas Mueller, Hemmen Sabir

**Affiliations:** 1 Department of Neonatology and Pediatric Intensive Care, Children’s Hospital, University Hopsital Bonn, 53127 Bonn, Germany; till.dresbach@ukbonn.de (T.D.); viktoria.rigoni@soft-consult.de (V.R.); anne.groteklaes@ukbonn.de (A.G.); a.mueller@ukbonn.de (A.M.); 2 Department of General Pediatrics, Neonatology and Pediatric Cardiology, University Children’s Hospital Duesseldorf, Medical Faculty, Heinrich Heine University, 40225 Duesseldorf, Germany; thomas.hoehn@uni-duesseldorf.de; 3 Department of Pediatrics I/Neonatology, University Hospital Essen, University of Duisburg-Essen, 45147 Essen, Germany; anja.stein@uk-essen.de (A.S.); ursula.felderhoff@uk-essen.de (U.F.-M.); 4 Centre for Translational Neuro- and Behavioral Sciences (C-TNBS), University Hospital Essen, University of Duisburg-Essen, 45147 Essen, Germany

**Keywords:** newborn, perinatal asphyxia, therapeutic hypothermia, treatment start, outcome

## Abstract

Background: Therapeutic hypothermia is the standard treatment for neonates with hypoxic–ischemic encephalopathy. Preclinical evidence indicates that the time to initiate therapeutic hypothermia correlates with its therapeutic success. This study aims to explore whether there is a correlation between the early initiation of therapeutic hypothermia and improved short-term neurological outcomes in cooled asphyxiated newborns. Methods: A retrospective analysis was conducted, involving 68 neonates from two different neonatal intensive care units. The impact of time to initiate treatment, time to reach the target temperature, and time between initiation and target temperature was correlated with short-term outcomes on MRI. Results: We did not find a significant difference between outcomes regarding the time to start treatment and the time to achieve the target temperature. Interestingly, neonates with a poor outcome were treated on average earlier than neonates with a favorable outcome but required more time to reach the target temperature. Additionally, the study results did not support the hypothesis that a shorter time to initiate treatment would lead to shorter times to achieve the target temperature. Conclusion: Based on our findings, it is recommended to prioritize a thorough evaluation of neonatal encephalopathy before initiating therapeutic hypothermia. Early initiation of treatment should be balanced with the time required for precise assessment to ensure better outcomes.

## 1. Introduction

Perinatal asphyxia leading to hypoxic–ischemic encephalopathy (HIE) is one of the main contributors to newborn mortality and the development of long-term neurological disabilities, such as seizures and cerebral palsy [1]. Therapeutic hypothermia (TH) for newborns born at or after 36 weeks and who suffer from moderate or severe HIE reduces the risk of death or disability at 18 months in high-income countries [2,3,4]. Therefore, the ILCOR guidelines have recommended TH (body temperature of 33.5 ± 0.5 °C, started within the first 6 h after birth for 72 h followed by controlled rewarming) as first-line therapy for the treatment of moderate to severe HIE following perinatal asphyxia in high-income countries since 2010 [5]. Animal studies have shown that the neuroprotective efficacy of the treatment is dependent on the time to initiate treatment following hypoxia–ischemia [6,7]. Whether this directly correlates with clinical findings remains under debate. However, it has been shown that cooling after 6 h is less beneficial compared to cooling within the first 6 h after birth in clinical studies [8], confirming animal findings [6]. As 6 h is a wide range to start treatment, we aimed to obtain evidence as to whether delaying the start of cooling and reaching the time to achieve the target temperature might impair outcomes. Since the implementation of TH, different studies on the initiation time of treatment have been published. In 2013, Thoresen et al. reported that cooling initiated before 3 h after birth was associated with improved long-term motor outcomes in a local cohort of 80 cooled neonates [9]. Unfortunately, even though the initiation time of cooling is a parameter that is easily adjustable, a new retrospective study from 2019 showed no correlation between the initiation time and MRI outcomes or long-term neurodevelopmental outcomes at 18 months [10]. Recently, Rao et al. presented a large cohort of cooled asphyxiated newborns [11]. In this cohort, time to reach the target temperature was also not associated with improved long-term outcomes.

The concept of “Time is brain” has spread widely in many industrialized countries within the last few years. This concept carries the risk of initiating treatment before confirming the diagnosis of moderate to severe HIE and might lead to the cooling of milder grades of encephalopathy. Whether cooling is beneficial for mild HIE is under current debate, as large, randomized controlled trials are yet to be conducted.

We aimed to evaluate the correlation between the initiation time of cooling and outcomes in a cohort of cooled asphyxiated newborns. We compared the outcomes of death and short-term MRI findings with the time to start cooling and the time to reach the target temperature in a local cohort of cooled asphyxiated newborns from two sites. Furthermore, we calculated the time between the start of cooling and reaching the target temperature and investigated the association with adverse outcomes.

## 2. Materials and Methods

### 2.1. Data Sources and Collection

Data from 68 cooled asphyxiated newborns were retrospectively collected. Newborns were treated in the neonatal departments of the University Hospital Essen and the University Hospital Düsseldorf between the years of 2008 and 2019. Ethical committees of both hospitals approved the retrospective data analysis (18-8191-BO/19-8556-BO, 2018-270-ProspDEuA). Studies on this cohort have already been published elsewhere [12,13].

Twenty-six newborns were born in-house and seventeen were transferred to the University Hospital Essen (*n* = 43). Fifteen newborns were born in-house and ten were transferred to the University Hospital Düsseldorf (*n* = 25).

The analyzed data were collected from newborns with a gestational age of ≥ 36 weeks who met the criteria for perinatal asphyxia, showed signs of mild–moderate or moderate–severe neonatal encephalopathy, and received therapeutic hypothermia postnatally starting within the first 6 h after birth for 72 h, maintaining a rectal temperature of 33.5 ± 0.5 °C.

The criteria for perinatal asphyxia were as follows: either (1) pH of ≤7.0 and/or base deficit (BD) ≥15 mmol/L taken from the umbilical artery or a blood sample taken immediately after birth or within the first hour of life; (2) APGAR score of ≤5 at 10 min; and/or (3) cardiopulmonary reanimation within the first 10 min of life. Additionally, the criteria included signs of mild, moderate, or severe encephalopathy using (1) the Sarnat stage (on a scale of 0–3) and/or (2) a pathological amplitude-integrated EEG pattern. Exclusion criteria were prematurity of <36 weeks of gestation, congenital malformations or intracerebral hemorrhage, and newborns ≥36 weeks who did not fulfill all required criteria.

We collected demographic and clinical data, such as birth weight, gestational age, worst pH, BD, lactate level, HIE Sarnat stage, APGAR score, time to start cooling, time to reach the target temperature, death, and MRI outcomes on days 7–14. MRI was performed with a 3 Tesla MR scanner (Magnetom Skyra, Siemens Healthcare, Erlangen, Germany). We defined the worst pH as the lowest pH before starting TH treatment. According to the in-house standard operating procedures, the time to start cooling was ≤6 h, and the rewarming process started after 72 h of hypothermia, increasing the temperature by 0.5 °C per hour. All newborns were treated according to in-house clinical standards, and the decision to treat was made by the local teams on clinical service at the time of treatment.

### 2.2. Outcome Definition

Since standardized long-term outcomes (Bayley Scales of Infant Development) were not available in our cohort, we used an established short-term outcome parameter, an established MRI score. Barkovich et al. first described the scoring system, which categorizes the outcome as good versus adverse depending on no, mild, moderate, or severe lesions in four different brain areas [14]. A favorable outcome was defined as a basal ganglia/watershed score ≤ 2, while an adverse outcome was considered as >2 points. The Barkovich score has been shown to significantly correlate with long-term outcomes in cooled asphyxiated newborns [15].

We defined a favorable outcome as no death and a positive MRI outcome and an adverse outcome as death or a poor MRI score.

### 2.3. Data Analysis

We conducted a comparative analysis using SPSS (Version 28, Chicago, IL, USA). A two-sample independent Mann–Whitney U-Test was used for non-parametric data. The variables included birthweight, gestational age, pH, BE, lactate level, HIE score, APGAR score at 10 min, death, time to start cooling, and time to reach the target temperature. Additionally, we performed a multivariate logistic regression analysis to examine the correlation of independent variables with the dependent variable outcome (normal outcome vs. death or abnormal MRI score). Independent variables included birthweight, gestational age, pH, BE, lactate level, 10 min APGAR score, birthplace (inborn vs. outborn) HIE score, time to start cooling, time to reach the target temperature, and time between start and reaching target temperature for all outcomes, as well as separately for survivors only. As we were interested in whether the early initiation of cooling was associated with better outcomes, we performed the regression analysis separately for newborns being treated with time to start cooling < 45 min and time to reach the target temperature ≤ 120 min. A *p*-value of <0.05 was considered significant. Descriptive data are presented as the median (range).

## 3. Results

### 3.1. Data Description

The statistical analysis included data from 68 newborns, comprising 35 females (51.47%) and 33 males (48.53%). Descriptive data for the two defined groups, consisting of 51 newborns with good outcomes and 17 newborns with adverse outcomes, are presented in Table 1.

For the good outcome group, the median (range) birthweight was 3160 g (1745–5050), while the adverse outcome group had a median birthweight of 3300 g (2100–4180). The median (range) gestational age was 272 days (242–291) for the good outcome group and 282 days (247–290) for the adverse outcome group.

In the good outcome group, the median (range) pH, BE, and lactate levels were 6.86 (6.49–7.23), 21 mmol/L (8.2–38), and 12 mmol/L (2.3–28). In the adverse outcome group, the median (range) pH level was 6.8 (6.4–7.14), the BE was 23 mmol/L (10.00–33.6), and the lactate level was 13.5 mmol/L (1.5–30). In the good outcome group, 31 newborns were inborn and 20 were outborn. In the adverse outcome group, 10 newborns were inborn and 7 were outborn. In the good outcome group, 33.3% of newborns showed mild signs of HIE (*n* = 17 mild, *n* = 25 moderate, and *n* = 9 severe (Sarnat stage)). In the adverse outcome group, only moderate and severe HIE stages were observed (*n* = 5 moderate and *n* = 12 severe Sarnat stage). In the adverse outcome group, nine newborns died, while there were no deaths in the good outcome group. The median (range) APGAR score at 10 min was 7 (0–10) for the good outcome group and 4 (0–10) for the adverse outcome group.

**Figure 1:**
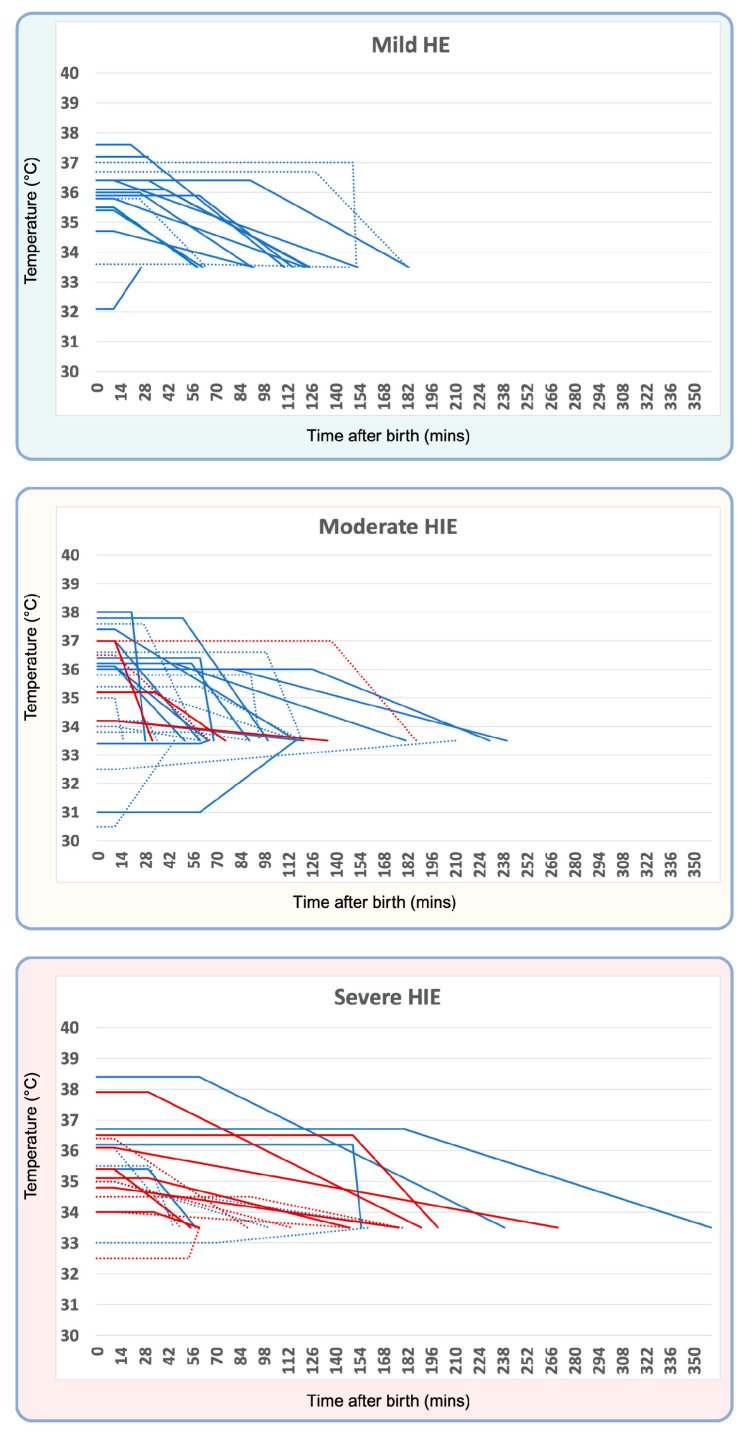
Temperature curves of individual patients. Patient cohorts divided by severity of encephalopathy (mild, moderate, or severe). Dotted lines represent outborn patients; blue lines represent patients with good outcomes, red lines represent patients with adverse outcomes (death or poor MRI outcome).

The median time to start cooling was 10 min, with a range of 10–150 min in the adverse outcome group. In the good outcome group, the median time to start cooling was 30 min, with a range of 10–180 min. Although the median time to start treatment was longer in the good outcome group, the time to reach the target temperature was longer in the adverse outcome group (median time to reach the target temperature = 110 min in the good outcome group vs. 135 min in the adverse outcome group). The median time to reach the target temperature after the initiation of cooling was 55 min (range 10–200 min) for the good outcome group. As the median time to reach the target temperature was later in the adverse outcome group, the results are similar for the time to reach the target temperature after the initiation of cooling (78 min (range 10–260) in the adverse outcome group). Individual patient data dependent on HIE severity (mild/moderate/severe) are presented in Figure 1.

### 3.2. Data Analysis

We observed that APGAR scores at 10 min were significantly lower in neonates from the adverse outcome group (*p* < 0.001). We did not observe significant differences between pH levels between groups. There was a significant difference in HIE grades (*p* < 0.001) between the two groups. Higher HIE grades were found in the adverse outcome group. The birthweight, gestational age, BE, and lactate levels were not significantly different between the groups. Regarding our key question, we observed that the time to start cooling, time to reach the target temperature, and time between start and target temperature did not differ significantly between the outcome groups (*p* = 0.502/*p* = 0.421/*p* = 0.192).

Regression analysis showed no significant influence of early initiation of cooling (<45 min (*p* = 0.777)), early time to reach the target temperature (≤120 min (*p* = 0.594)), pH, lactate level, BE, 10 min APGAR score, and birthweight on the dependent outcome variables. Additionally, the regression showed no influence of the time between the start of cooling and reaching the target temperature (*p* = 0.392).

As significance was not proven, we concluded that the time to start treatment, as well as the time to reach the target temperature, could not be used as an outcome predictor. We reevaluated our results, just containing the survivors, to prove that the outcome for survivors could neither be predicted through the time to start cooling <45 min (*p* = 0.891) nor the time to reach the target temperature ≤120 min (*p* = 0.229) (Table 2 and Table 3).

We analyzed whether outborn patients had different outcomes and times to start treatment and times to achieve the target temperature. We were surprised not to find a correlation with outcomes dependent on birthplace (Table 4).

## 4. Discussion

The retrospective data analysis conducted in this study aimed to investigate a potential association between the time to start therapeutic hypothermia treatment, the time to reach the target temperature, and the time to reach the target temperature after the initiation of treatment with short-term MRI findings at days 7–14. We found no significant association between the time to start cooling and different outcomes (death or adverse MRI outcome). Additionally, we observed no significant difference in the time to reach the target temperature across the different short-term outcome groups. Furthermore, regression analyses showed no significant association between time to reach the target temperature and short-term outcomes. To account for the discrepancy between the adverse outcome group having a shorter time to start cooling but a longer time to reach the target temperature, we introduced the concept of time to reach the target temperature after treatment initiation. Unfortunately, we did not find a significant correlation between the time and short-term outcomes.

Animal experiments of hypoxic–ischemic encephalopathy have indicated that the early initiation of therapeutic hypothermia improves histological and functional outcomes [6,7]. In these models, a step-wise increase in time between experimental hypoxia–ischemia and cooling treatment led to a stepwise reduction in neuroprotection. This knowledge is guided by primary clinical treatment protocols, which are still current clinical practice, and indicates that cooling needs to be started within the first 6 h after birth in the presence of moderate to severe neonatal encephalopathy following perinatal asphyxia. In the large, randomized controlled cooling trials, the concept of early initiation of cooling treatment could not be assessed, as parental consent had to be obtained before the initiation of cooling. Therefore, these trials were unable to answer the question of whether the early initiation of cooling would be beneficial compared to later cooling. In contrast to preclinical studies, injury severity and the on-set time of hypoxia–ischemia are unknown at delivery. In newborn rats, cooling was not efficient after severe hypoxia–ischemia, even when started immediately after the insult. In this model, delaying hypothermia (>6 h) led to an increase in brain injury [6]. Transferring this preclinical knowledge to the clinical scenario can sometimes be challenging. This might explain why therapeutic hypothermia has limited efficacy in reducing death or major disability in survivors (overall risk reduction of 25% in the large, randomized cooling trials performed before 2010 [16]). The latest study from 2017 analyzing the efficacy of cooling within the first 6 h after birth in high-income countries found that 72 h of therapeutic hypothermia (33–34 °C) reduced the rate of death and major disability in survivors by 29% [17]. This is a direct improvement to the large, randomized controlled trial performed by the same study group in 2005, where the rate of death and major disability in survivors was 44% [3]. This improvement was due to lower rates of severe encephalopathy and the earlier initiation of cooling.

In a clinical study, Laptook et al. found that delayed cooling (6–24 h post delivery) did not significantly improve death or disability compared to non-cooling. However, they found that cooling initiated between 6 to 24 h after birth, as opposed to non-cooling, led to a 76% likelihood of experiencing some decrease in death or disability, and a 64% likelihood of experiencing at least a 2% decrease in death or disability at 18 to 22 months [8].

The concept of “time is brain” has been assessed in local cohort studies as the original randomized controlled cooling trials are not helpful in assessing this question. Thoresen et al. showed in 2013 that the early initiation of cooling was associated with better long-term motor outcomes [9]. They reported in their local cohort of 80 newborns with moderate to severe HIE that cooling after 3 h was less effective than cooling within the first 3 h after birth in survivors. However, they did not find a difference in the combined outcome of death or severe disability between the early and late cooling groups. A second study including 49 cooled asphyxiated newborns found that cooling within the first hour of life (*n* = 20) was associated with a decreased seizure burden [18]. However, almost 40% of infants from the late cooling group were outborn and had to be transferred before the diagnosis of neonatal encephalopathy was made. Therefore, the primary management of these patients might have been different from that of inborn patients. Even though 40% of our analyzed patients were also outborn, we did not find an association of short-term findings dependent on birthplace. Studies by Guillot et al. and Gilmore et al. also investigated the association between early cooling (within the first 3 h after birth) and improvement of outcomes in their local cohorts [10,19]. Even though outcome parameters were not similar between the two studies (early (MRI) vs. late (neurodevelopmental testing) outcomes), neither study found an improvement in outcome parameters after the early initiation of cooling. The latest study by Rao et al. included 500 cooled asphyxiated newborns being enrolled in the HEAL study [11]. The authors also did not find an improvement in long-term neurodevelopmental outcomes depending on the time to reach the target temperature. They found that newborns with severe HIE reached the target temperature earlier compared to infants with moderate HIE. However, this did not have an impact on the outcome. Taken together, the available retrospective data analyses are not consistent regarding the “time is brain” concept. This might be due to differences in local cohorts and the different management of individual patients. A prospective data collection is therefore urgently needed. Very early initiation of therapeutic hypothermia before detailed clinical examination might interfere with the correct diagnosis of neonatal encephalopathy and might explain why therapeutic hypothermia is only beneficial in some of the treated newborns. The inclusion of newborns with mild HIE will change the results and will make it difficult to compare data from different cohorts. Detailed neurological examination and, if feasible, the usage of amplitude-integrated electroencephalography (aEEG) should be the gold standard compared to very early decision-making. Describing and reporting the detailed entry criteria for cooling treatment is very valuable. However, this information is sometimes missing when performing retrospective analyses. We are also not able to report why in our study newborns with mild HIE were included, as this was due to individual treatment decisions. In addition, outcome measures often differ between studies. We used Barkovich MRI scoring to assess outcomes in our cohort. This MRI scoring system has been established in non-cooled asphyxiated newborns but has proven validity in cooled asphyxiated newborns in predicting long-term outcomes [15]. We recently confirmed that Barkovich scoring is associated with 2-year long-term outcomes in a local cohort of cooled asphyxiated newborns [20]. In general, the Bayley Scales of Infant Development assessed at 18–24 months are used in many publications as the gold standard of neurodevelopmental outcomes. However, it has been shown that 18–24 months Bayley scores correlate poorly with long-term outcomes at school age [21]. Therefore, assessing long-term outcomes in retrospective studies would be the ultimate aim.

There are limitations to our study. First, as previously mentioned, only assessing short-term outcomes is a major limitation. However, we have shown that Barkovich MRI scoring results correspond to 18–24 month Bayley results [20]. Therefore, we believe that our findings correspond with longer-term outcomes. The aim of future studies should be to test children at different ages to assess the validity of the Bayley Scales in our local cohorts. Second, the decision to start cooling was based on individual clinical decisions and cannot be retrospectively reviewed. As mentioned above, the correct timing of the diagnosis of neonatal encephalopathy is very important. Third, we included 17 newborns with mild HIE grades in our analysis. All infants were cooled based on the local treatment guidelines, which recommended treatment of moderate to severe encephalopathies. Why individual treatment decisions were made to include newborns with mild HIE cannot be verified, as this is not feasible due to the retrospective nature of the study. Analyzing the results separately based on the severity of HIE did not change our results, however.

Therapeutic hypothermia became a clinical standard almost 15 years ago. However, it is surprising that clinical guidelines are often lacking, and treatment protocols vary between different countries. In Germany, currently, a nationwide treatment guideline is missing. Recently, the German hypothermia register was established, aiming to provide data on cooled asphyxiated newborns. The register will have the possibility of providing data on the current management of the affected newborns and might help to develop prospective data analysis and investigative research questions in clinical trials.

## 5. Conclusions

In conclusion, we found that the time to initiate treatment and reach the target temperature is not associated with short-term MRI pathology. Additionally, our cohort did not provide any evidence that a shorter duration to initiate therapeutic hypothermia and achieve the target temperature offers a benefit to short-term outcomes. Therefore, we recommend a precise clinical evaluation of neonatal encephalopathy before the initiation of TH.

## Figures and Tables

**Table 1:** Descriptive data of the analyzed cohort. Bold indicating significant differences between the groups.

	Good Outcome (*n* = 51)	Adverse Outcome ^1^ (*n* = 17)	*p*-Value
Gender (*n*, %)	*n* = 25 female (49.02%) *n* = 26 male (50.98%)	*n* = 10 female (58.82%) *n* = 7 male (41.18%)	0.495
Birthweight (gram, median (range))	3160 (1745–5050)	3300 (2100–4180)	0.767
Gestational age (days, median (range)) Birthplace (*n*, %)	272 (242–291) Inborn *n* = 31 (60.78%) Outborn *n* = 20 (39.22%)	282 (247–290) Inborn *n* = 10 (58.82%) Outborn *n* = 7 (41.18%)	0.105 0.891
pH (median (range))	6.86 (6.49–7.23)	6.8 (6.4–7.14)	0.057
BE (median (range))	21 (8.2–38)	23 (10.00–33.6)	0.373
APGAR score 10 min (median (range))	7 (0–10)	4 (0–10)	**<0.001**
HIE grade (*n* = mild, *n* = moderate, *n* = severe)	17 = mild (33.33%) 25 = moderate (49.02%) 9 = severe (17.65%)	0 = mild (0.0%) 5 = moderate (29.41%) 12 = severe (70.59%)	**<0.001**
Death (*n*, %) Seizures during treatment aEEG time to normal trace (hours, median (range))	*n* = 0 (0.0%) Yes = 21 No = 30 9 (1–168)	*n* = 9 (52.941%) Yes = 14 No = 3 73 (1–360)	**<0.001** **0.002** **<0.001**
Time to start cooling (minutes, median (range))	30 (10–180)	10 (10–150)	0.502
Time to reach the target temperature (minutes, median (range))	110 (10–360)	135 (32–270)	0.421
Time to reach the target temperature after the initiation of cooling (minutes, median (range))	55 (5–200)	78 (6–260)	0.192

^1^ Poor MRI outcome/+death.

**Table 2:** Division of analyzed cohort.

	Time to Start Cooling < 45 min	Time to Start Cooling ≥ 45 min
Good Outcome (*n* = 51 (%))	*n* = 30 (59%)	*n* = 21 (41%)
Adverse Outcome ^1^ (*n* = 17)	*n* = 12 (70%)	*n* = 5 (30%)
Death (*n* = 9)	*n* = 7 (78%)	*n* = 2 (22%)

^1^ Poor MRI outcome or death.

**Table 3:** Division of analyzed cohort.

	Time to Reach the Target Temperature ≤ 120 min	Time to Reach the Target Temperature > 120
Good Outcome (*n* = 51)	*n* = 32 (63%)	*n* = 19 (37%)
Adverse Outcome ^1^ (*n* = 17)	*n* = 8 (47%)	*n* = 9 (53%)
Death (*n* = 9)	*n* = 4 (44%)	*n* = 5 (56%)

^1^ Poor MRI outcome or death.

**Table 4:** Outcome divided by birthplace.

	Inborn (*n* = 41)	Outborn (*n* = 27)
Good Outcome	*n* = 31 (76%)	*n* = 20 (74%)
Adverse Outcome ^1^	*n* = 10 (24%)	*n* = 7 (26%)
Death	*n* = 4 (10%)	*n* = 5 (18%)
Time to start cooling, minutes (median, (range))	30 (10–180)	30 (10–50)
Time to reach the target temperature, minutes (median, (range))	116 (26–360)	101 (10–210)

^1^ Poor MRI outcome or death.

## Data Availability

All data can be obtained from the corresponding author. The data are not publicly availabile due to legal reasons.
